# Health Literacy: An Interactive Outcome Among Secondary Students in Beijing

**DOI:** 10.3928/24748307-20201117-01

**Published:** 2021-01-11

**Authors:** Shuaijun Guo, Xiaoming Yu, Elise Davis, Rebecca Armstrong, Lucio Naccarella

## Abstract

**Background:**

Health literacy enables a person to make good decisions regarding health care, disease prevention, and health promotion to maintain and improve health. Although health literacy research in China has gained increasing attention in recent years, most existing studies focus on adults rather than adolescents. In addition, little theory-driven empirical research has been conducted to fully understand the relationship among health literacy, its influencing factors, and health outcomes scored on a skills-based health literacy instrument.

**Objective:**

This study applied Manganello's framework to investigate how health literacy was related to its antecedents and health status in secondary students in Beijing, China.

**Methods:**

A cross-sectional study was conducted with 650 students in Years 7 to 9 (age 11–17 years) from four secondary schools. Students completed a self-administered questionnaire based on Manganello's health literacy framework, which measured key upstream determinants, including health literacy and self-report health status. Health literacy was measured on an 8-item skills-based instrument that assesses a person's ability to find, understand, appraise, and communicate health information in everyday life (scores range from 0–37). Descriptive statistics and path analysis were conducted to investigate the mediating role of health literacy in predicting health status.

**Key Results:**

Overall, the average scores of students' health literacy was 26.37 (±5.89). Manganello's framework was supported by the data collected (*χ^2^*/df = 2.049, *p* = .001, comparative fix index = 0.966, root mean square error of approximation = 0.041). Personal self-efficacy (*r* = 0.11, p = .007), social support (*r* = 0.18, p < .001), and school environment (*r* = 0.27, *p* < .001) predicted health literacy, which in turn predicted students' health status (*r* = 0.12, *p* = .005).

**Conclusions:**

Adolescent health literacy is not only a person's capability to protect health, but also an interactive outcome with the broader environment. Promoting health literacy could be a useful strategy to improve health status for adolescents; however, a holistic approach is needed to increase students' self-efficacy, promote social support, and create positive school environments to achieve optimal health literacy and health outcomes. **[*HLRP: Health Literacy Research and Practice.* 2021;5(1):e1–e14.]**

**Plain Language Summary:**

We investigated how health literacy was related to its influencing factors and health status among secondary students in Years 7 to 9 in Beijing, China. Students with low self-efficacy, low social support, and low perceptions of positive school environment were more likely to have low health literacy, which in turn predicted poor health status.

Health literacy, defined as “the ability to engage with health information and services,” is a personal asset that enables a person to make healthy decisions regarding health care, disease prevention, and health promotion to maintain and improve health (Nutbeam, 2008; World Health Organization, 2015). The relationship between health literacy and health outcomes has been well established, with people who have limited health literacy experiencing more health-compromising behaviors, poorer health status, and higher health care cost (Berkman et al., 2011; McDonald & Shenkman, 2018). From a health promotion perspective, improving health literacy at an early age is important to adolescent health and empowerment both now and in the future (Manganello, 2008).

Although adolescence is commonly viewed as a healthy time of life, adolescents are facing unprecedented health challenges in the 21st century (Patton et al., 2016). First, noncommunicable diseases such as mental health disorders and substance use disorders are becoming the dominant health problems of this age group. Second, the high prevalence of health-risk behaviors (e.g., physical inactivity, unhealthy eating) among adolescents suggests an urgent need for developing early interventions to prevent long-term health consequences in adulthood (Fleary et al., 2018). Third, adolescents are growing up in a digital world and face significant challenges when accessing online health information and making health decisions (Levin-Zamir & Bertschi, 2018). An effective strategy to reduce health disparities arising from the above three challenges is promoting adolescent health literacy, which has been well documented in empirical studies, particularly in school settings (Hagell et al., 2015; Perry, 2014). For example, the Building Wellness program conducted in the United States (Diamond et al., 2011), a youth health literacy curriculum targeting youth with low income in grades 3 to 8, showed positive outcomes (e.g., improved healthy behaviors) in preparing youth to be active participants for their own health care.

Adolescent health literacy is a continuum over time, following a developmental trajectory from infancy to adolescence, with more health knowledge and skills acquired as a person grows (Abrams, et al. 2009). Specifically, there are six unique characteristics (the 6 “D's”) of health literacy for this age group (Bröder et al., 2019). The first “D” is “differential epidemiology and health perspectives,” which means that adolescents are experiencing a unique pattern of health, illness, and disability. Although they may partly suffer from similar diseases as adults, some diseases are highly age- or development-specific. The second “D” is “demographic patterns and health inequalities,” which notes that adolescents are especially vulnerable to health inequalities and are the age group with the highest risk of poverty. The third “D” is “developmental change and socialization process,” as adolescents are experiencing a life stage in which physical, emotional, cognitive, and social development processes take place. The fourth “D” is that adolescents are more “dependent” on their parents, friends, and peers when making health decisions. The fifth “D” is “democratic citizenship and active participation,” because this age group has the right to be informed and to participate actively in their own health. The last “D” is for “digitization,” as many adolescents are growing up in highly digitized and media-saturated settings. National and international studies have shown that low health literacy is prevalent in adolescents, ranging from 34% in the US to 93.7% in China (Guo, 2018; Sansom-Daly et al., 2016). Given that health literacy is an important and modifiable determinant of health (Velardo & Drummond, 2017), addressing low health literacy in adolescents is essential to maximize future health and social outcomes.

Understanding and investigating how adolescent health literacy can be improved is a burgeoning research area around the world, including in China (Bröder et al., 2018; Bröder et al., 2017; Peralta et al., 2017). The earliest government document regarding adolescent health literacy is the “Chinese Primary and Secondary School Health Education Guideline” (hereafter referred to as “the Guideline”), which was issued by the Ministry of Education in 2008 (Ministry of Education of the People's Republic of China, 2008). Improving students' health literacy was specified as one goal for primary and secondary school health education. Health literacy in the Guideline was conceptualized as having three domains: conceptual knowledge and attitudes (71 items), behavior and lifestyles (48 items), and health-related skills (40 items) (Ministry of Education of the People's Republic of China, 2008). Due to the impact of this political document, health literacy instruments in China mainly focus on health knowledge and practices (Ye et al., 2014; Yu et al., 2012), rather than health skills such as communicating and appraising health information (Guo, Armstrong, et al., 2018). This makes measurement of health literacy not equivalent with, and results non-comparable between, China and other countries.

Health literacy is a multidimensional concept that needs to be understood in a particular context and for a specific content among a specific population (Nutbeam et al., 2018). In the present study, we defined health literacy as a person's ability to find, understand, appraise, and use health information in everyday life and apply it in school settings. Health literacy is operationalized as having three domains (Nutbeam, 2000): the functional domain focuses on a person's basic skills in reading and writing health information; the interactive domain emphasizes extracting information from different sources and communicating skills to protect health; and the critical domain represents more advanced skills like appraising health information and applying it into practice. In addition, Manganello's (2008) health literacy framework was slightly modified and employed in the present study because this theoretical framework fully illustrates how adolescent health literacy relates to its influencing factors and health outcomes. Manganello's health literacy framework was informed by the ecological theory and Nutbeam's three-domain health literacy model (Bronfenbrenner, 1977; Manganello, 2008; Nutbeam, 2000). It has three main modules: (1) upstream factors that may influence health literacy (e.g., socio-demographics); (2) components that comprise the health literacy construct; and (3) downstream health outcomes (e.g., health status) that may contribute to health literacy (Manganello, 2008).

Currently, most health literacy studies in China focus on adults rather than adolescents (Tang et al., 2019; Y. Wu et al., 2017; Xie et al., 2019), but if they do then they mainly use measures based on health knowledge and practices (Ye et al., 2014; Yu et al., 2012; Zhang et al., 2019); however, it remains unclear how skills-based health literacy instruments perform in Chinese adolescents. Furthermore, little theory-driven empirical research has been conducted. Without the use of a theoretical framework, most existing research fails to provide a holistic understanding of health literacy, resulting in a simplistic understanding of the relationships between health literacy and either key upstream factors or health-related outcomes (Lam & Yang, 2014; Ye et al., 2014; Yu et al., 2012; Zhang et al., 2019). To fill the above two research gaps, we developed the research question for the present study: “What are the relationships among health literacy, its upstream influencing factors, and downstream health outcomes, based on a skills-based health literacy instrument?” Recently, such a skills-based instrument has been developed to measure health literacy in Chinese adolescents (Guo, Davis, et al., 2018). The present study aims to apply this skills-based health literacy instrument and investigate how adolescent health literacy is related to key upstream factors and health status, using Manganello's health literacy framework as a guide (Manganello, 2008).

## Methods

### Settings and Sample

A cross-sectional study was designed to recruit adolescents from secondary schools in two districts (one high socio-economic status and one low socio-economic status) of Beijing, China, using convenience sampling. Schools were chosen because they are the most common places where adolescents spend most of their time during the day. It is, therefore, feasible and achievable to recruit large samples in a short time. Two secondary schools in each district were selected based on previous research partnerships and appropriate survey timing (class time, class break time, or lunch time). Thereafter, two intact classes in each year level (Years 7, 8, and 9) (children age 11–17 years) were chosen, with the number of students in each class ranging from 20 to 35. Students in each class were asked to complete a self-administered questionnaire. In total, 650 students participated in the study, which is a sample size considered acceptable for path analysis (Golob, 2003). Data collection was undertaken in November 2015 and was approved by the ethics committee of Peking University (Ethics ID: IRB00001052-15024) and The University of Melbourne (Ethics ID: 1442884).

### Questionnaire

Based on Manganello's health literacy framework (Manganello, 2008), we designed a questionnaire to measure students' health literacy, key upstream factors, and health status. Details of each subscale are presented in **Table 1**.

***Intrapersonal factors.*** The factors collected were sociodemographics and personal self-efficacy. Socio-demographics included age, gender (male/female), ethnicity (Han Chinese or ethnic minorities), year level in school (Year 7, 8, or 9), family structure (intact families/other types), and family affluence level (low, medium, or high) (Liu et al., 2012). Personal self-efficacy was measured by the General Self-Efficacy Scale (GSES) (Schwarzer et al., 1997), which is a 10-item scale that assesses personal belief in the ability to cope with a variety of challenges in life. Respondents indicated their level of agreement on a 4-point scale (1 = *not at all true*, 4 = *exactly true*). The GSES is available in Chinese and has strong structural validity and excellent internal consistency (Schwarzer et al., 1997). The GSES total score range is 10 to 40, with higher scores indicating higher levels of self-efficacy.

***Interpersonal factors.*** Interpersonal factors were assessed using the Multidimensional Scale of Perceived Social Support (MSPSS) (Chou, 2000), a 12-item scale that measured a person's perceived support from family, friends, and significant others. Respondents answered each item on a 7-point Likert scale (1 = *very strongly disagree*, 7 = *very strongly agree*). The MSPSS has been validated in Chinese adolescents, showing high internal consistency, satisfactory concurrent validity, and construct validity (Chou, 2000). The MSPSS total score range is 12 to 84, with higher scores reflecting higher levels of social support.

***Environmental factors*.** School environment was assessed by the School Environment Scale (SES), which was derived from the Communities That Care Youth Survey (Glaser et al., 2005). The SES is comprises 10 items measuring students' subjective feelings about opportunities and rewards for pro-social involvement at school. Respondents indicated their level of agreement with each statement on a 4-point Likert scale (1 = *strongly disagree*, 4 = *strongly agree*). In the present study, the SES showed high internal consistency (Cronbach's α = 0.88) and satisfactory construct validity (comparative fit index [CFI] = 0.095 to 0.996, root mean square error of approximation [RMSEA] = 0.048∼0.053). The SES total score range was 10 to 40, with higher scores suggesting stronger bonds of attachment to school.

Community environment was assessed by the Community Environment Scale (CES), which measured respondents' subjective feelings of their neighborhood environment such as cleanliness and safety (Gray & Sanson, 2005). The CES consisted of nine items in three domains: neighborhood livability, neighborhood facilities, and traffic. Participants answered each item on a 5-point scale (0 = *do not know,* 1 = *strongly disagree*, 4 = *strongly agree*;). The CES showed adequate internal consistency (Cronbach's α = 0.84) and satisfactory construct validity (exploratory factor analysis indicated a three-factor construct and explained 67.78% of the total variance, with factor loadings greater than 0.48 on all items) in the present study. The CES total score range was 0 to 36, with higher scores indicating a more livable and supportive community.

***Health literacy.*** Health literacy was assessed using the eight-item Health Literacy Assessment Tool (HLAT-8), which measures a person's ability to access, understand, evaluate, and communicate health information in everyday life (Abel et al., 2015). The HLAT-8 total score range was 0 to 37, with higher scores indicating higher levels of health literacy. The HLAT-8 has been validated in Chinese secondary students, showing satisfactory reliability and strong validity (Guo, Davis, et al., 2018).

***Health status.*** This was assessed with a widely used general self-report health question (“In general, would you say your health is?” 1 = *poor*, 5 = *excellent*) (Waters et al., 2001). This single question has demonstrated strong predictive validity with objective indicators of health and mortality (Haddock et al., 2006).

### Data Analysis

All statistical analyses were conducted with SPSS (version 23) and with IBM AMOS Statistics (version 23). Descriptive statistics were used to examine participants' socio-demographic variables and each measured scale (frequency/percentage, mean, median). Univariate analysis (*t-*test, analysis of variance, nonparametric test) and correlation analysis (Pearson and Spearman correlation analysis) were conducted to examine the associations among key upstream factors, health literacy, and health status. Path analysis was then conducted using the maximum likelihood method. Model fit was examined with the relative chi-square goodness-of-fit statistic (χ^2^/*df*), CFI, Tucker and Lewis's Index of Fit (TLI), and RMSEA. An acceptable model fit was considered when the χ^2^/*df* statistic ≤3, CFI values ≥0.95, TLI values ≥0.95, and RMSEA values ≤0.08 (Hu & Bentler, 1999).

The percentages of missing items for the GSES, MSPSS, SES, CES, HLAT-8, and health status ranged from 0.9% to 1.8%, 0.9% to 2.0%, 0.9% to 1.7%, 2.5% to 2.9%, 0.2% to 0.6% and 0%, respectively. Due to a small proportion of missing values, individual mean substitution was conducted for non-response items in each self-report scale. Data normality was assessed using skewness and kurtosis values. Results showed that only scores on self-efficacy, health literacy, and school environment were distributed normally, whereas scores on social support and community environment showed non-normal distribution.

## Results

### Participant Characteristics

As shown in **Table 2**, the mean age of participants was 13.42 years (range, 11–17 years), with a standard deviation of 1.01. Students' gender and year level were evenly distributed. Almost one-quarter of students came from families with low-affluence and one-third self-reported poor or fair health status. The average scores of health literacy in our sample were 26.37 (±5.89).

### Relationships Among Upstream Factors, Health Literacy, and Health Status

**Table 3** shows that there are differences in scores of self-efficacy, social support, school environment, community environment, and health literacy by gender, year level, family structure, and family affluence level. Overall, students were more likely to have high scores on self-efficacy, social support, school environment, community environment, and health literacy if they came from families that were intact with high-affluence families. Correlation analysis showed that students' health literacy was positively correlated with self-efficacy, social support, school environment, community environment, and health status (*r* = 0.21–0.57, *p* < .01) (**Table 4**).

### The Mediating Role of Health Literacy in Predicting Health Status

**Figure 1.**
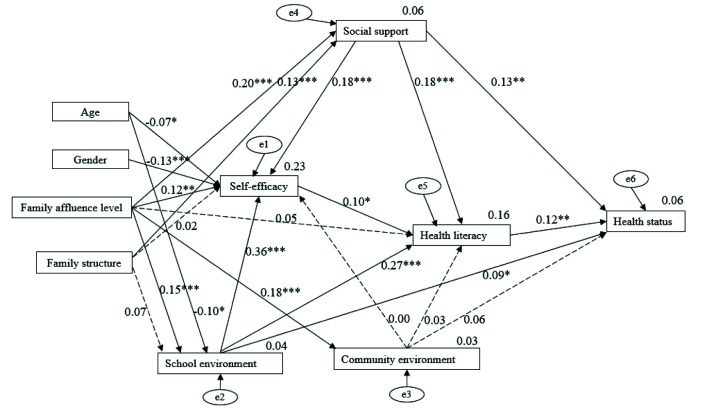
The original path model for Beijing secondary school students. Coefficients are standardized path coefficients. The error term (e1–e6) is the residual term, representing the margin of error within a statistical model and providing an explanation for the difference between the results of the model and actual observed results. Overall model fit, χ^2^/*df* (23, *N* = 625) = 15.043, *p* < .001, comparative fit index = 0.597, root mean square error of approximation = 0.150 (90% confidence interval [0.136, 0.164]). For tests of significance of individual paths, ^*^p < .05, ^**^p < .01, and ^***^p < .001.

**Figure 2.**
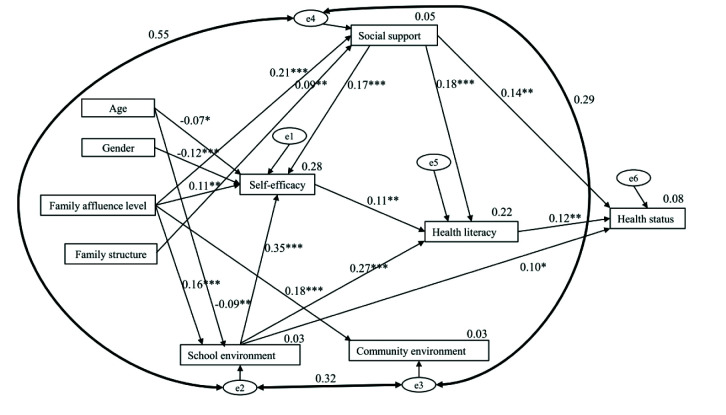
The trimmed path model for Beijing secondary school students. Coefficients are standardized path coefficients. The error term (e1–e6) is the residual term, representing the margin of error within a statistical model and providing an explanation for the difference between the results of the model and actual observed results. Overall model fit, χ^2^/df (26, *N* = 625) = 2.049, *p* = .001, comparative fit index = 0.966, Tucker and Lewis's Index of Fit = 0.941, root mean square error of approximation = 0.041 (90% confidence interval [0.025, 0.057]). For tests of significance of individual paths, ^*^p < .05, ^**^p < .01, and ^***^p < .001.

After univariate and correlation analyses, all significant independent variables related to health literacy and/or health status were considered for next-step path analysis. The original path model demonstrated poor data fit (**Figure 1**): χ^2^/*df* (23, *n* = 625) = 15.043, *p* < .001, CFI = 0.597, TLI = 0.211, RMSEA = 0.150 (90% confidence interval [0.136, 0.164]), but the path from health literacy to health status was significant (*r* = 0.12, *p* = .006). Examination of modification indices suggested that the model fit could be improved by connecting errors between social support and school environment, between school environment and community environment, and between social support and community environment (**Table A**), represented by the bold, double-headed arrows in the trimmed model (**Figure 2**). These modifications were made based on ecological theory (Wharf Higgins et al., 2009), which suggests that social support, school environment, and community environment all influence students' health literacy. The final trimmed path model demonstrated excellent data fit: χ^2^/*df* (26, *n* = 625) = 2.049, *p* = .001, CFI = 0.966, TLI = 0.941, RMSEA = 0.041 (90% confidence interval [0.025, 0.057]).

In the final trimmed path model, there were significant and direct paths from self-efficacy (*r* = 0.11, *p* = .007), social support (*r* = 0.18, *p* < .001), and school environment (*r* = 0.27, *p* < .001) to health literacy, and health literacy (*r* = 0.12, *p* =.005) to health status. Additional significant paths are shown in **Table B**. Based on the squared multiple correlation coefficients (*r*^2^), the final trimmed model explained 28% of the variance in self-efficacy, 22% of the variance in health literacy, and 8% of the variance in health status.

## Discussion

The current study tested a cross-sectional path model linking upstream factors through health literacy to health status among secondary school students in Beijing, China. Specifically, there were three key findings from the path analysis. First, socio-demographics were not directly associated with health literacy; however, an indirect effect was observed through personal self-efficacy, social support, and perceptions of school and community environment. Second, self-efficacy, social support, and perceptions of school environment were independently associated with health literacy. Third, health literacy was found to be associated with self-reported health status.

Inconsistent with previous research using a similar analytic approach (Gwynn et al., 2016; Xie et al., 2019), we did not find direct paths from socio-demographics to health literacy. Although students' family affluence level was associated with health literacy in our univariate analysis, the association was attenuated when considering personal self-efficacy, social support, and school environment in the path model. One possible explanation for the difference between our findings and previous research is due to the specific socio-demographic characteristics of our sample, which was a younger population with a narrower age range and the same educational level. In addition, the homogeneity (ethnicity, family structure, and socio-economic status) of our sample was higher than that of previous studies, which assessed students from different cultural backgrounds (Chang, 2011; Ghaddar et al., 2012;A. Wu et al., 2010). Another reason could be that students' self-efficacy, social support, and school environment are more proximal and direct predictors of health literacy than socio-demographics. However, due to the cross-sectional nature of our data, there is a need for future research using longitudinal data and more representative samples to differentiate the direct and indirect path from socio-demographics to health literacy.

Consistent with Manganello's health literacy framework, we found that students' health literacy was affected by self-efficacy, social support, and the school environment (Manganello, 2008). This empirical finding also supports the validity of previous similar theoretical frameworks that advocate an ecological perspective of adolescent health literacy (Wharf Higgins et al., 2009). This suggests that low health literacy is not only an individual person's issue, but that it results from close interactions with the broader environment. For example, adolescent health literacy appears to depend more heavily on social support and available resources around them than on personal self-efficacy. Compared with adults, adolescents have less well-developed cognitive ability (Borzekowski, 2009). Therefore, they are more likely to seek support from peers, parents, and others when they encounter health problems. School environment is another significant influencing factor for adolescent health literacy because it is the primary place where students develop and promote health literacy (St Leger, 2001). The quality of the school environment is, therefore, likely to directly affect students' access to health knowledge, attitudes towards changing unhealthy behaviors, and mastery of health skills (Kolbe, 2005). Enhancing personal self-efficacy may not be enough to counter low health literacy in adolescents. Instead, the intervention strategy for promoting health literacy should integrate programs that aim to improve students' social support and to create supportive school environments using a holistic approach to eventually improving adolescent health.

This study also extends our understanding of the relationship between health literacy and health status in school-aged adolescents. Health literacy was found to mediate the relationship between upstream factors and health status, suggesting that it is possible to improve students' health status through enhancing health literacy for those with low self-efficacy, social support, and perceptions of positive school environment. School-based interventions from an ecological perspective have been widely accepted and well documented as a useful strategy to improve student health overall (Hornby, 2016; Wang et al., 2017). Health literacy, as an interactive outcome between a person's capacity and the broader environment, could be an integral part of the holistic approach to maximizing the effectiveness of school-based interventions. Interventions for students with poor health status should not only enhance personal self-efficacy through approaches like “action planning” (Williams & French, 2011), increase social support such as instrumental and motivational support (Beets et al., 2010), and promote school physical/social environment like playground improvements (Bonell et al., 2013), but also improve students' health literacy such as by delivering skills-based health curricula (Hubbard & Rainey, 2007). In addition, empirical evidence suggests that building health-literate organizations is effective to promote equitable access and engagement and support adolescents to participate in healthy decisions regarding their health and social wellbeing (World Health Organization, 2015). Therefore, schools and community health organizations can be also be health literate themselves to meet the needs of all adolescents with different health literacy skills, thus contributing to better health outcomes for this young generation (Peralta et al., 2017; Peralta & Rowling, 2018).

## Strengths and Limitations

One strength of the present study is that we employed a skills-based, valid, and multi-dimensional instrument to measure adolescent health literacy rather than focusing on health knowledge and behaviors. The other strength is the use of Manganello's health literacy framework as a guide to understand the full relationship among health literacy, key upstream factors, and health status (Manganello, 2008). This enhanced the rigor, transparency, and clarity of the current research.

However, this study is not without limitations. First, this study only used cross-sectional data to examine the pathways from key upstream factors through health literacy to health status at a single time-point. Longitudinal studies or randomized controlled trials are needed in the future to confirm the mediation effect of health literacy on health status. Second, the convenience sampling may limit the generalizability of our findings. We recruited students from four secondary schools in a large city where the ability of participants to access good education might be much higher than the general population. Future studies are recommended to recruit adolescents from a wider range of socio-demographic backgrounds. Third, we did not include “mass media” as an environmental factor in our theoretical framework. Given that adolescents are growing up in an increasingly media-saturated and digitized world and are encountering a large proportion of health-related messages electronically (Bröder et al., 2019; Levin-Zamir & Bertschi, 2018), there is a need for future research to explore how mass media influences adolescent health literacy, as well as how it interacts with other upstream factors (e.g., self-efficacy, social support, school environment) to influence adolescent health literacy. Fourth, self-report bias may exist because we only used a single item measurement scale for the outcome “health status.” Future research work using more robust outcome measures is warranted. Finally, we conducted path analysis, rather than structural equation modeling, to investigate the mediating role of health literacy in predicting health status. Path analysis was considered more appropriate in this study because there were a high number of outcome variables, making the structural equation modeling approach more complex to analyze and interpret.

## Conclusion

Adolescent health literacy is not only an adolescent's personal asset/capability to protect health, but also an interactive outcome with the broader environment. We found that Manganello's health literacy framework was supported by the empirical data related to health status (Manganello, 2008). Adolescent health literacy mediated the association between a set of ecological factors (self-efficacy, social support, and school environment) and health status. Promoting health literacy could be a useful strategy to improve adolescents' overall health status, but a holistic approach is needed to increase students' self-efficacy, promote social support, and create positive school environments to achieve optimal health literacy and health outcomes.

## Figures and Tables

**Table 1:** Measurement of Key Upstream Factors, Health Literacy, and Health Status

**Construct**	**Measure**	**Example Item**	**Scoring**	**Coding**	**α**
Intrapersonal factors					
Age	A single-item measurement of students' age	How old are you?	Age was self-reported by students themselves	Continuous	-
Gender	A single-item measurement of students' sex	Are you male or female?	Gender was self-reported by students themselves	Binary: male; female	-
Ethnicity	A single-item measurement of students' ethnicity	What is your ethnicity?	Ethnicity was self-reported by students themselves	Binary: Han; ethnic minorities	-
Year level	A single-item measurement of students' year level	What year level are you in at school?	Year level was self-reported by students themselves	Categorical: Year 7; Year 8; Year 9	-
Family structure	A single-item measurement of students' family structure	Think of where you live most of the time. Who usually lives there with you?	Family structure was self-reported by students themselves. Intact families were defined as those in which participants indicated residing in a household with both biological parents, whereas other types of families were defined as those in which participants indicated residing in a household with either one of their parents, foster parents, step parents, a relative or who were living in a shared care institution	Binary: Intact; other types	-
Family affluence level	The 4-item Family Affluence Scale (FAS)	Do you have your bedroom for yourself?own	Students self-reported family affluence in terms of the number(s) of cars, computers, bedrooms and family holidays. The FAS total score range was 0–7	Ordinal: low (0–3); medium (4–5); and high (6–7)	-
Self-efficacy	The 10-item General Self-Efficacy Scale (GSES)	I can always manage to solve difficult problems if I try hard enough	Students self-reported personal belief in the ability to cope with a variety of challenges in life. The GSES total score range was 10–40	Continuous	0.89

Interpersonal factors Social support	The 12-item Multidimensional Scale of Perceived Social Support (MSPSS)	My family really tries to help me	Students self-reported support from family, friends and significant others. The MSPSS total score range was 12–84	Continuous	0.93

Environmental factors					
School environment	The 10-item School Environment Scale (SES)	I feel safe at my school	Students self-reported feelings about opportunities and rewards for pro-social involvement at school. The SES total score range was 10–40	Continuous	0.88
Community environ ment	The 9-item Community Environment Scale (CES)	This is a safe neighborhood	Students self-reported feelings of their neighborhood environment such as cleanliness and safety. The CES total score range was 0–36	Continuous	0.84

Health literacy					
Health literacy	The 8-item Health Literacy Assessment Tool (HLAT-8)	When I have questions on diseases or health problems (e.g. headache, back pain, sport injury), I know where I can find information on these issues	Students self-reportedtheir ability to access, understand, evaluate, and communicate health information in everyday life. The HLAT-8 total score range was 0–37	Continuous	0.79

Health outcomes					
Health status	A single-item measurement of students' health status	In general, would you say your health is?	Health status was self-reported by students themselves	Ordinal: fair or poor; good; excellent or very good	-

**Table 2:** Participants' Socio-demographics and Descriptive Statistics of Measured Scales (*N* = 650)

**Characteristic**	***n* (%)**

Gender	
Male	357 (54.9)
Female	293 (45.1)

Year level	
7	232 (35.7)
8	215 (33.1)
9	203 (31.2)

Ethnicity	
Han Chinese	617 (94.9)
Ethnic minorities (Hui, Chaoxian, Menggu)	33 (5.1)

Family structure	
Intact family	552 (84.9)
Other types	97 (14.9)
Missing	1 (0.2)

Family affluence level	
Low	179 (27.5)
Medium	296 (45.5)
High	169 (26.0)
Missing	6 (0.9)

Age, years^a^	13.42 ± 1.01

Self-efficacy^a^ (score range 10–40)	26.85 ± 6.37

Social support^a^ (score range 12–84)	65.73 (54, 73)

School environment^a^ (score range 10–40)	30.48 ± 5.59

Community environment^a^ (score range 0–36)	26 (24, 30)

Health literacy^a^ (score range 0–37)	26.37 ± 5.89

Health status	
Fair or poor	224 (34.5)
Good	227 (34.9)
Excellent or very good	199 (30.6)

Note. IQR = interquartile range.

a Continuous variables are described by mean ± *SD* or median (interquartile range).

**Table 3:** Univariate Analysis of Self-Efficacy, Social Support, School Environment, Community Environment, and Health Literacy by Participant Characteristics

**Characteristic**	**Self-Efficacy**		**Social Support**		**School Environment**		**Community Environment**		**Health Literacy**	
	**Mean ± *SD***	***p* Value**	**Median (IQR)**	***p* Value**	**Mean ± *SD***	***p* Value**	**Median (IQR)**	***p* Value**	**Mean ± *SD***	***p* Value**

Gender										
Male	27.49 ± 6.24	.004^*^	65 (54, 73)	.954	30.39 ± 5.72	.646	26 (23, 31)	.569	26.40 ± 6.16	.881
Female	26.06 ± 6.44		66 (53, 74)		30.60 ± 5.4		26 (24, 29.25)		26.33 ± 5.56	

Year level										
7	27.96 ± 6.43	.003^*^	66 (53, 74)	.132	31.40 ± 5.38	< .001^*^	26 (23, 31)	.622	26.78 ± 5.75	.117
8	26.54 ± 6.61		66 (55.25, 73)		30.65 ± 5.82		26 (23.90, 30)		26.59 ± 5.41	
9	25.91 ± 5.86		62 (50, 73)		29.27 ± 5.38		26 (24, 29)		25.67 ± 6.48	

Ethnicity										
Han Chinese	26.89 ± 6.28	.595	65.23 (54, 73)	.985	30.55 ± 5.54	.213	26 (24, 30)	.399	26.37 ± 5.89	.926
Ethnic minorities (Hui, Chaoxian, Menggu)	26.13 ± 7.91		67 (52.25, 74.25)		29.28 ± 6.36		28.50 (23.75, 30)		26.27 ± 5.96	

Family structure										
Intact family	27.09 ± 6.36	.023^*^	66 (56, 74)	.002^*^	30.67 ± 5.54	.045^*^	26 (24, 30)	.105	26.49 ± 5.79	.215
Other types	25.47 ± 6.29		59 (49, 72)		29.43 ± 5.79		26 (22, 29)		25.68 ± 6.47	

Family affluence level										
Low	25.20 ± 6.27	< .001^*^	60 (49, 69.50)	< .001^*^	29.41 ± 5.68	< .001^*^	25 (22, 27)	< .001^*^	25.30 ± 5.51	.012^*^
Medium	26.96 ± 6.17		67 (56, 74)		30.45 ± 5.43		26 (24, 30)		26.72 ± 5.95	
High	28.47 ± 6.50		69 (57.61, 78)		31.83 ± 5.48		28 (25, 32)		26.96 ± 5.82	

Note. IQR = interquartile range.

* *p* < .05

**Table 4:** Correlation Analysis Among Self-Efficacy, Social Support, School Environment, Community Environment, Health Literacy, and Health Status

**Variable**	**Self-Efficacy**	**Social Support**	**School Environment**	**Community Environment**	**Health Literacy**	**Health Status**
Self-efficacy	1.000	-	-	-	-	-
Social support	0.446^**^	1.000	-	-	-	-
School environment	0.475^**^	0.572^**^	1.000	-	-	-
Community environment	0.283^**^	0.375^**^	0.389^**^	1.000	-	-
Health literacy	0.319^**^	0.432^**^	0.427^**^	0.253^**^	1.000	-
Health status	0.289^**^	0.266^**^	0.212^**^	0.210^**^	0.276^**^	1.000

Note.

** *p* < .01.

**Table A:** Modifications for Health Status Path Model in Secondary Students in Beijing, China

**Model**	***χ*^2^**	***df***	***p* Value**	**CFI**	**RMSEA [90% CI]**
Original model	345.987	23	< .001	0.597	0.150 [0.136, 0.164]
Remove nonsignificant paths	354.022	29	< .001	0.594	0.134 [0.122, 0.147]
Modification 1 (path e2<--->e4)	134.114	28	<.001	0.868	0.078 [0.065, 0.091]
Modification 2 (path e2<--->e3)	109.082	27	< .001	0.898	0.070 [0.056, 0.084]
Modification 3 (path e3<--->e4)	53.274	26	.001	0.966	0.041 [0.025, 0.057]
Final model	53.274	26	.001	0.966	0.041 [0.025, 0.057]

Note. Path e2<--->e4: path was made between the error of school environment and the error of social support; Path e2<--->e3: path was made between the error of school environment and the error of community environment; Path e3<--->e4: path was made between the error of community environment and the error of social support. *χ*^2^ = conventional chi-square fit statistic (under maximum likelihood estimate); CFI = comparative fit index; CI = confidence interval; *df* = degree of freedom; RMSEA = root mean square error of approximation.

**Table B:** Individual Parameter Estimation for Health Status Path Model in Secondary Students in Beijing, China

**Parameter**	**Coefficient**	**Standardized Coefficient**	**Standard Error**	***p* Value**
Social support <--- family structure	3.800	0.090	1.367	.005
Social support <--- family affluence level	1.994	0.206	0.378	< .001
School environment <--- age	−0.525	−0.095	0.179	.003
School environment <--- family affluence level	0.558	0.157	0.140	< .001
Self-efficacy <--- school environment	0.395	0.347	0.047	< .001
Self-efficacy <--- social support	0.072	0.172	0.017	< .001
Self-efficacy <--- age	−0.455	−0.072	0.216	.035
Self-efficacy <--- gender	−1.558	−0.122	0.434	< .001
Self-efficacy <--- family affluence level	0.459	0.113	0.141	.001
Health literacy <--- self-efficacy	0.099	0.109	0.037	.007
Health literacy <--- school environment	0.277	0.268	0.047	< .001
Health literacy <--- social support	0.070	0.184	0.016	< .001
Health status <--- school environment	0.018	0.099	0.009	.038
Health status <--- social support	0.009	0.137	0.003	.004
Health status <--- health literacy	0.021	0.122	0.008	.005
Community environment <--- family affluence level	0.694	0.181	0.151	< .001
